# Association between Blood Donation and Malignant and Benign Tumour Risk: A Population-Based Study of 3.4 Million Participants in China

**DOI:** 10.1155/2022/7647431

**Published:** 2022-07-08

**Authors:** Shu Su, Ting Ma, Yang Sun, Lingxia Guo, Xiaodong Su, Wenhua Wang, Xinxin Xie, Liqin Wang, Lili Xing, Leilei Zhang, Shiyi He, Jiangcun Yang, Lei Zhang

**Affiliations:** ^1^Department of Transfusion Medicine, Shaanxi Provincial People's Hospital, Xi'an, China; ^2^Clinical Research Management Office, The Second Affiliated Hospital of Chongqing Medical University, Chongqing, China; ^3^China-Australia Joint Research Centre for Infectious Diseases, School of Public Health, Xi'an Jiaotong University Health Science Centre, Xi'an, China; ^4^Data Center, Shaanxi Provincial People's Hospital, Xi'an, China; ^5^Planning Development and Information Office, Health Commission of Shaanxi Province, Shaanxi Province, China; ^6^The Department of Medical Record Management, Shaanxi Provincial People's Hospital, Xi'an, China; ^7^Artificial Intelligence and Modelling in Epidemiology Program, Melbourne Sexual Health Centre, Alfred Health, Melbourne, Australia; ^8^Department of Epidemiology and Biostatistics, College of Public Health, Zhengzhou University, Zhengzhou 450001, Henan, China; ^9^Central Clinical School, Faculty of Medicine, Monash University, Melbourne, Australia

## Abstract

This study aims to identify the relationship between blood donation and malignant and benign tumour hospitalization risk. The cohort study was constructed in Shaanxi, China, to include blood donors and match nonblood donors one-to-one by gender, age, and county of residence. The study compared the hospitalization records of two groups from 2012 to 2018. A log-binomial regression model was used to estimate the relative risk (RR) of tumour risk between donors and nonblood donors among different age groups. A total of 1,625,599 donors were recruited (including 968,823 males) and compared with the matched nonblood donor group. Significantly lower risk of malignancy in males was found among donors (adjusted RR: 0.82, 95% CI: 0.75–0.92). Lower risks for specific types of tumours among donors were observed, including liver (0.42, [0.28–0.67]), lung (0.74, [0.59–0.87]), lymphoma (0.75, [0.62–0.85]), and oesophagus (0.55, [0.41–0.72]). However, the risk of brain cancer was higher among male donors (RR 1.19 [1.06–1.29]). Among female donors, lower risk of liver (0.57, [0.42–0.79]) and oesophagus malignancy (0.73, [0.62–0.88]) was observed. For benign tumours, male donors have a lower risk of benign skin tumour (0.79, [0.62–0.94]) and hemangioma and lymphangioma (0.75, [0.51–0.89]), while female donors have a lower risk in hemangioma and lymphangioma (0.65, [0.44–0.83]). We also found that the risk decreased with age among donors in the prevalence of tumours compared to that in nonblood donors (*p* < 0.05). Blood donation appears to be significantly associated with various tumour risks among both males and females. Overall, the risk of tumours decreased more substantially with age in blood donors compared with nonblood donors. Further research is warranted to investigate the impact of ‘health donor effects' on these findings.

## 1. Background

Blood donation is known to benefit blood recipients, especially patients who are in accidents or surgeries that require them to urgently receive large volumes of blood. To ensure safe blood use, the World Health Organization (WHO) encouraged volunteer blood donation instead of commercial blood donation in 2020 [1]. However, it is controversial whether blood donations have an impact on the general health of blood donors; no studies have thoroughly investigated it. Previous studies have reported that blood donation can decrease the risk of lung cancer, stroke, and red blood cell disorders and that frequent whole-blood donation can impact the cardiovascular morbidity of whole-blood female donors [2, 3]. However, these studies did not include large sample sizes or long-term follow-up with their participants. Few related studies have investigated the association between donation and tumours though malignant tumours are the leading cause of death in China population, which shows the necessity to conduct this study.

The impacts of blood donation on tumour prevalence are difficult to investigate, as the progression of the disease is linked to ageing and requires long-term follow-up to provide strong evidence. Although previous studies have shown low rates of cancer incidence and all-cause mortality among blood donors [4–6], suggesting that blood donation is not harmful, the potentially deleterious effects of a blood donation may be masked by the healthy lifestyles and good health of the donor population [7, 8]. Apart from immediate complications, such as syncope and local bleeding, the short- and long-term health effects of blood donations have yet to be thoroughly investigated.

The controversy surrounding this issue may be due to limitations from the involvement of participants with known high-risk health behaviours, such as smoking or alcohol addiction, and family histories of cancer [9–11]. Thus, the present study excluded participants with the abovementioned high-risk factors and attempted to explore the association between blood donation and tumour risk. Certain current and advanced treatments have used bloodletting therapy to treat specific diseases [12–22]. However, the malignant tumour has a lower risk in the healthy population in China. Although malignant tumours are some of the most fatal diseases in the world and benign tumours can also develop into cancer, no study has investigated the relationship between blood donation and benign tumour risk. This study attempted to provide more suggestions for people health who donated blood. By using a large cohort study of Shaanxi blood donors and individuals one-to-one matched with the general population, this study aimed to identify the role of blood donation in both malignant and benign tumour risk.

## 2. Methods

### 2.1. Data Source

#### 2.1.1. The Shaanxi Blood Donation Database Is a Computerized Combined Donation and Transfusion

Register from Shaanxi Province, which is located in northwest China, with a permanent resident population of approximate 39 million in 2019: Over the period 1998 to 2018, 3.4 million individuals donated blood voluntarily, and their relevant blood information was collected in the Shaanxi Blood Donor Database. Linking it with the province-wide Shaanxi Electronic Health Records (EHR) and Centralised Hospital Medical Records (CHMRs) provides an unprecedented opportunity to investigate the potential benefits and adversity for any clinically diagnosed diseases in donors (compared with nonblood donors) potentially as a result of blood donation, in a natural and representative population cohort [23].

The database covered all ten prefectures (Xi'an, Xianyang, Tongchuan, Baoji, Weinan, Yan'an, Hanzhong, Yulin, Ankang, and Shangluo) in Shaanxi. Each blood station at the prefecture-level adopted a standardised blood collection and information management system. Information from individual prefectural blood stations was collected and exported by the system administrators and subsequently integrated into a large dataset at the provincial level. After a recent update, the database includes information on 1.7 million blood donors who have donated blood since 1998 and 2018. Using unique personal national registration numbers assigned to all residents in the database, it can be linked to nationwide population, death, and migration registers, thus ensuring complete follow-up of all study participants. The Shaanxi Blood Donor's Database was subsequently integrated with Shaanxi resident's Electronic Health Records (EHR), administered by Shaanxi Provincial Health Planning Policy Evaluation and Information Center. EHR was a key digital health initiative implemented nationwide by the Chinese government in 2009 and widely recorded in Shaanxi since 2012 [24]. The database can be linked to inpatient, outpatient, cause of death, and cancer registers, allowing long-term follow-up for a range of health outcomes from EHR. The outcome indicator of the study was the onset of the new tumour cases.

### 2.2. Study Design

**Figure 1:**
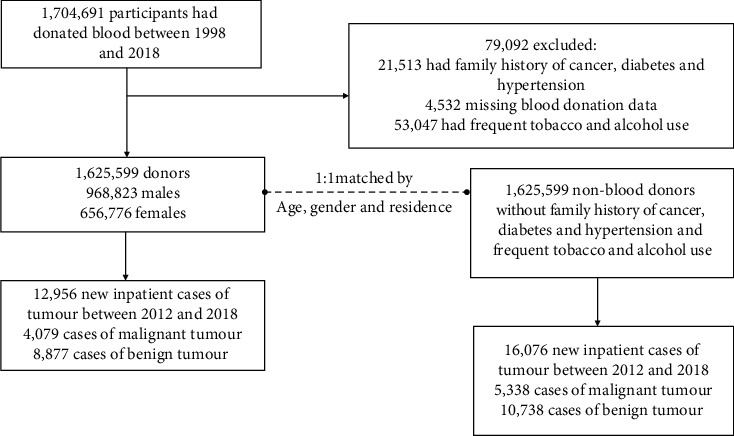
Study flowchart for the identification of malignant and benign tumour cases.

In this cohort, we obtained all the health information data from both blood donors and nonblood donors with one-to-one matching demographic characteristics (gender, age, and residence) to investigate the onset and reoccurrence of a wide spectrum of both malignant and benign tumours defined by ICD-10. The tumour incident was recorded during 2012–2018 extracted from inpatient record, as it is accurate and tumour case was mainly from hospitalization diagnosis. The matching was conducted by random sampling without replacement of individuals without any history of blood donation from the EHR and comparing the three characteristics with those from the blood donor cohort. Both participants from blood donors and nonblood donors who have tumour-related high risky behaviours were excluded, including smoking and drinking, and participants with a family history of cancers were excluded. In this cohort study, we used medical and sociodemographic data obtained at the time EHR was built. Among the 1,704,691 registered blood donors, patients with a family history of cancer, diabetes, or hypertension (*n* = 21,513), missing blood donation data (*n* = 4,532), or frequent smoking and alcohol abuse (*n* = 53,047) were excluded (Figure 1). Because of early symptoms, donors with an incipient malignancy are likely to stop donating blood, which could lead to health donor effects. We, therefore, carried out the following analysis to only include the patients who have donation activity six months before tumour diagnosis.

### 2.3. Statistical Analysis

We stratified the case by malignant tumours and benign tumours among males and females. We selected the top ten highest incident malignant tumours from China's cancer statistics to do the comparison. Regarding the benign tumours, we selected the top ten highest incident benign tumours from donors to do the comparison as there is no rank for a benign tumour in China. Four log-binomial regression models to identify female and male malignant and benign tumours, respectively, were built to analyse the associations between tumour incidence and blood donation in the respective exposure windows. The regression was adjusted by gender, sex, age, ethnicity, occupation, education, and marital status. We also examined the tumour risk associated with age as a continuous variable to test for a linear trend in both genders, respectively. According to the China blood donation guidelines, the criterion of the age group for blood donation is 18–55 so we divided the age categories into 18–25, 26–35, 36–45, 46–55, and >55 years old five categories; the age was used as participants entered the cohort. The model compared the tumour incidence between donors and nonblood donors among four age groups. Relative risk (RR) was used as an estimate of relative risks. Descriptive and inferential statistical analyses were performed. The mean, median, and IQR were used to summarise numerical variables, whereas frequencies and percentages were used to describe categorical variables. All statistical analyses and data handling were conducted with computer software (SAS version 9.4., SAS Institute). Informed consent was obtained when the donor donated blood.

Ethical approval for the study was obtained from the institutional review board of the People's Hospital of Shaanxi Province (No: 2020-R002). The study was preregistered at China National Medical Research Register and Chinese Clinical Trial Registry (http://www.chictr.org.cn/index.aspx); the registration number is MR-61-21–011750 and ChiCTR2200055983, respectively.

The waiver of participants' consent was obtained given that all the participants in the cohort are deidentified, and the study design is observed. We have already signed the agreement of waiving consent from Shaanxi Provincial People's Hospital, Shaanxi Provincial Blood Center, and Shaanxi Health Information Center according to the 25th clause of the Declaration of Helsinki.

## 3. Results

### 3.1. Baseline Characteristics

The study finally included 1,625,599 donors (3,038,420 total blood donations) and 1,625,599 nonblood donors. The 1,625,599 blood donors included 968,823 males and 656,776 females. 1,625,599 nonblood donors without a family history of cancer, diabetes, and hypertension from EHR, who did not smoke frequently and did not drink alcohol excessively, were selected as controls. From 2012 to 2018, there were 12,956 new inpatient cases of tumour among blood donors (4,079 cases of malignant tumour, 8,877 cases of benign tumour), and 16,076 new inpatient cases of tumour from nonblood donors (5,338 cases of malignant tumour, 10,738 cases of benign tumour).

Baseline characteristics were displayed in Table 1. Of the 1,625,599 blood donors, 968,823 blood donors were male while 656,776 were female. The median (IQR) age was 35(28–44) years. Most of the blood donors (70.68%) came from Middle Shaanxi, 24.00% from South Shaanxi, and 5.32% from North Shaanxi. 56.21% of the blood donors had received the education of junior high school or below, 61.04% of the blood donors were peasants, and 66.40% of the blood donors were married. Among the people who donated blood more than two times, the time interval of blood donation was 1772 (672–3001) days.

### 3.2. Malignant Tumour Prevalence

**Figure 2:**
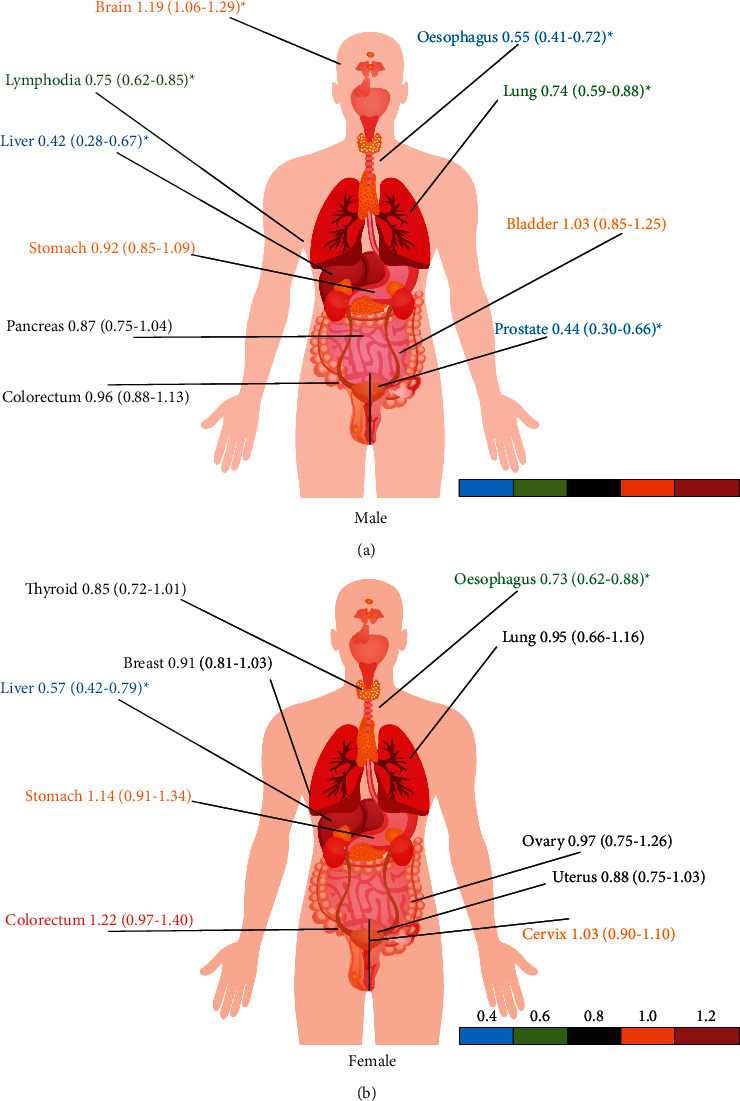
Comparing the relative risk of malignant tumours between blood donors and nonblood donors (2012–2018).

There was a significant difference in the hospitalization prevalence of malignant tumours between male donors and nonblood donors (*P* < 0.05, RR = 0.82, 95% CI (0.75–0.92)). The total prevalence of malignant tumours in male blood donors was 0.21%, while that in nonblood donors was 0.28%. There was also a significant difference in the total hospitalization prevalence of malignant tumours between female blood donors (0.31%) and nonblood donors (0.40%) (*P* < 0.01). The highest prevalence of malignant tumours in males is lung cancer, in both blood donors and nonblood donors (0.03% and 0.05%, respectively). The highest prevalence of malignant tumours in female blood donors and nonblood donors was breast cancer (0.08% and 0.09%, respectively). Blood donors with lower risk of malignant tumours included lung cancer (0.74, [0.59–0.88]), liver cancer (0.42, [0.28–0.67]), oesophagus cancer (0.55, [0.41–0.72]), prostate cancer (0.44, [0.30–0.66]), and lymphoma (0.75, [0.62–0.85]) compared with nonblood donors. Brain cancer was the only malignant tumour in donors who had a higher risk than nonblood donors (1.19, [1.06–1.29]). Regarding female donors, the risk of oesophageal cancer (0.73, [0.62–0.88]) and liver cancer (0.57, [0.42–0.79]) in blood donors was significantly lower than that in nonblood donors (*P* < 0.05) (Figure 2).

As shown in Table 2, the relative risk of malignant tumours among blood donors and nonblood donors of different genders decreased with age. The risk of malignant tumour in male blood donors aged 46–55 and over 55 years was significantly lower than that in nonblood donors (0.79, [0.58–0.89]) and 0.62, [0.49–0.77], respectively), and the risk of malignant tumour in female blood donors over 55 years old was also significantly lower than that in nonblood donors (0.57, [0.43–0.94]). The ratio in both genders showed a significant downward trend in both genders (p-trend < 0.05).

### 3.3. Benign Tumour Prevalence

**Figure 3:**
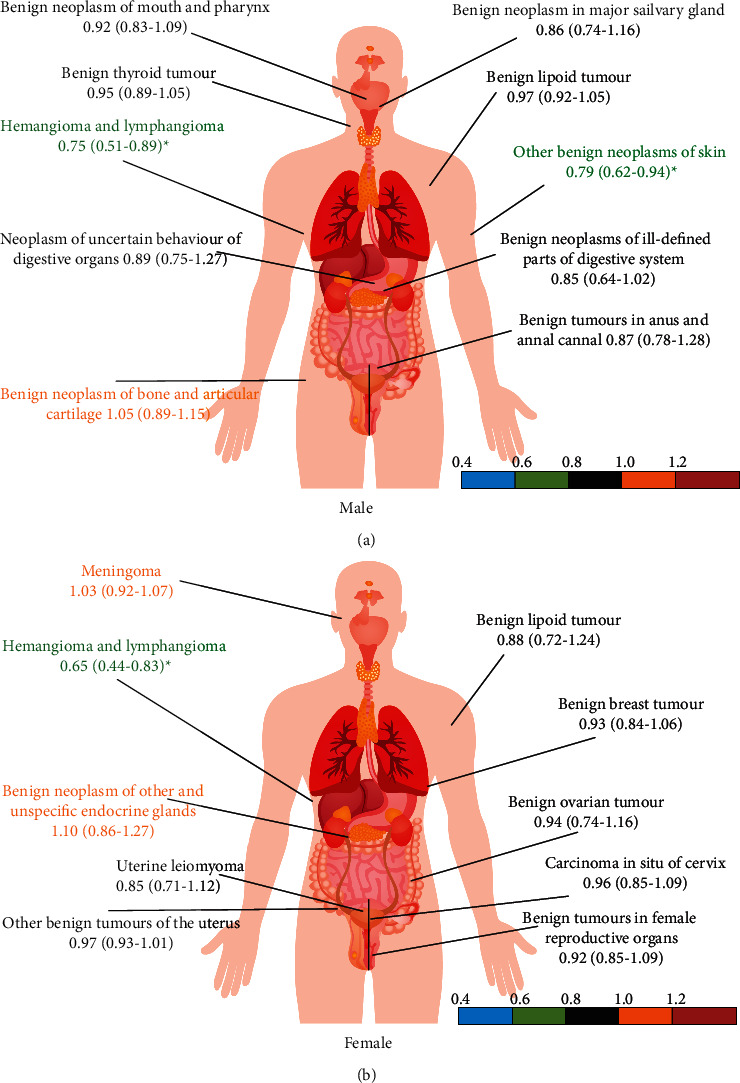
Comparing the relative risk of benign tumours between blood donors and nonblood donors (2012–2018).

For males, the prevalence of benign neoplasm in the major salivary gland was the highest in both blood donors and nonblood donors, and there was no statistical difference in the relative risk. The risk of hemangioma and lymphangioma and other benign neoplasms of the skin was significantly lower than that in nonblood donors (0.75, [0.51–0.89] and 0.79, [0.62–0.94], respectively). For females, the prevalence of uterine leiomyoma was the highest among blood donors (2216, 0.34%) and nonblood donors (2299, 0.35%). The total benign tumour prevalence was significantly lower in donors compared with nonblood donors (0.65, [0.44–0.83]) (Figure 3).

As shown in Table 3, the risk of benign tumour hospitalization in male blood donors over 55 years old was significantly lower than that in nonblood donors (0.69, [0.50–0.94]). The risk of benign tumour hospitalization in female donors aged 46–55 and over 55 years was also significantly lower than that in nonblood donors (0.78, [0.59–0.95] and 0.52 [0.36–0.81], respectively). Similarly, the p-trend test showed there was a decreased trend in risk between donors and nonblood donors with age increase (*p* < 0.05).

## 4. Discussion

Using a large database of Shaanxi Province blood donors covers the blood donor database for twenty years and covered 95% of Shaanxi Province. With complete follow-up with virtually no loss to follow-up, our study provides comprehensive evidence of associations between blood donation and increased or decreased risk of malignant and benign tumours among both genders. Our study found lower risk with blood donation remained for malignant tumours of the liver and oesophagus among the participants and gender differences were observed in blood donors. To our knowledge, this is the first evaluation study to identify blood donation and tumour risk in China.

Five solid malignant tumours were found associated with lower risk among male participants while two malignant tumours were related to lower risk among female donors. The result is consistent with the finding in the previous study that the incidence of cancer risk has declined among blood donors [5]. In the terms of genders, it seems that males have benefited more than females, as in China male malignant tumour incidence was higher than that in females according to the Chinese cancer statistic report. Also, some studies have found the risk of males has decreased with donating blood but is not observed among females. It may be because menstruation among females plays a role in it, as some studies have reported the underlying possible mechanism including the relationship between iron overload and blood donation. Several reasons may explain why our prevalence was lower than the national statistic. We have excluded the people with a high-risk factor of tumour incident, including people with long-term smoking and alcohol addiction who may need further observation.

Our study is the first one exploring the relationship between blood donation and benign tumours. The association between blood donation and the benign tumour was observed in hemangioma and lymphangioma among males and females. Consistently, males with the same benign tumour have lower risk than those with malignant tumours, and also a similar study was reported in Sweden [6]. As a benign tumour has some chance to develop to be a malignant tumour, it is necessary to implement preventive interventions.

This study also reports the risk has decreased with the age increase in both benign tumours and malignant tumours when comparing donors and nonblood donors. It should be notable that iron accumulation is a long-term process. The relationship between ageing and blood donation effects requires further exploration. The benefit to population health may be explained by iron depletion, which catalyses the production of a range of reactive oxygen species and free radicals [25, 26]. Although low iron stores have been associated with decreased risk of cardiovascular disease [27–29], most recent studies, including a clinical trial where subjects with the peripheral arterial disease were randomly assigned to have their stored iron reduced through bloodletting [30, 31], have not resulted in any perceivable protection [32, 33]. High levels of iron may be associated with cancers of the lung, liver, colon, stomach, and oesophagus [25, 34]. In addition, while several reports have investigated the association between stored iron levels and cancer risk, as indicated by various laboratory markers, the results have varied considerably [35]. Studies have suggested that increased cell proliferation, which is itself a possible risk factor for cancer [36, 37] and is stimulated by the repeated removal of blood cells, may increase the risk of hematopoietic malignancies in blood.

Some limitations should be noted in the study. First, if the residents have left Shaanxi we cannot collect their medical records out of this province, so the result may be underestimated and the follow-up record may be incomplete. In 2018, the proportion of Shaanxi residents who live or work in other Chinese provinces was approximately 15% [23]. Second, the mortality database was not fulfilled as we only have the patients with death outcomes in the hospital but for the participants died at home we cannot label their death records. Moreover, the average age of participants was young and the expected life in Shaanxi was 77 years [38], so the death rate should be low among the participants. Third, the medical records were only extracted between January 2012 and December 2018, so the onset of disease earlier than this period cannot be included. Fourth, our study did not consider the impacts of blood types on diseases as the majority of the nonblood donors (approximately 80%) lacked blood type information. We note that the blood types may have some significant impacts on the incidence of diseases [39] and plan to further investigate this factor in the model to compare the health status of blood donors.

## 5. Conclusion

Our study suggests a consistent association between blood donation and both malignant and benign tumours, with uniformly lower risk in blood donors for tumour events. The long-term benefit of blood donation was observed among donors, especially for donors older than 55 (the upper age limit for blood donation in China). From the findings, it seems that male donors had an even greater difference in tumour risk between blood donors and nonblood donors than females. The clinical diagnosis and gender difference in population health associated with blood donation need further exploration.

## 6. Novelty and Impact Statements

Blood donation saves lives and is essential for clinical care. To our best knowledge, few studies investigate the association between blood donation and its subsequent impacts on malignant and benign tumour in a cohort study. We address this knowledge gap through a large database of Shaanxi Province blood donors; it includes donors during the past twenty years and seven years' follow-up of hospitalization.

## Figures and Tables

**Table 1:** Basic demographic characteristics of blood donors and nonblood donors in the study.

Participants	Nonblood donors	Donors
Total (n, %)	1625599	1625599
Gender		
Male	968823	968823
Female	656776	656776
Age, years	35 (28–44)	35 (28–44)
Region		
North Shaanxi (*n*, %)	86540	86540
Middle Shaanxi (*n*, %)	1148936	1148936
South Shaanxi (*n*, %)	390123	390123
Education		
Junior high and below	913711	897254
Senior high	443576	419499
University and above	65779	108228
Unknown	202533	200618
Occupation		
Student	54331	90144
Worker	178022	227108
Peasant	992334	856698
Self-employed	372667	431531
Unknown	28245	20118
Marriage		
Single	422656	433382
Married	1079398	1071802
Divorced	11379	19911
Unknown	112166	100504
Days between blood donations (if participants with more than one donation)	—	1772 (672–3001)

**Table 2:** Overall and age-stratified malignant tumour cases in blood donors versus nonblood donors among males and females.

Male (malignant)	Donors	Nonblood donors	Donors vs. nonblood donors RR	P-trend
18–25	185	217	0.97 (0.83–1.32)	*P* < 0.01
26–35	306	333	0.97 (0.81–1.21)	
36–45	580	728	0.90 (0.88–1.31)	
46–55	646	835	0.79 (0.58–0.89)^∗^	
>55	344	620	0.62 (0.49–0.77)^∗^	

Female (malignant)				
18–25	233	260	0.95 (0.79–1.19)	*P*=0.01
26–35	319	381	0.88 (0.74–1.11)	
36–45	757	837	0.91 (0.80–1.07)	
46–55	569	698	0.85 (0.70–1.13)	
>55	140	429	0.57 (0.43–0.94)^∗^	

**Table 3:** Overall and age-stratified benign tumour cases in blood donors versus nonblood donors among males and females.

Male (benign)	Donors	Nonblood donors	Donors vs. nonblood donors RR	P-trend
18–25	621	639	0.96 (0.88–1.09)	*P* < 0.01
26–35	683	719	0.94 (0.90–1.06)	
36–45	670	734	0.91 (0.87–1.04)	
46–55	396	491	0.87 (0.82–1.11)	
>55	227	338	0.69 (0.50–0.94)^*∗*^	

Female (benign)				
18–25	1757	1915	0.98 (0.95–1.09)	*P* < 0.01
26–35	1316	1682	0.89 (0.76–1.08)	
36–45	2365	2723	0.91 (0.82–1.06)	
46–55	723	1087	0.78 (0.59–0.95)^*∗*^	
>55	119	410	0.52 (0.36–0.81)^*∗*^	

## Data Availability

The data that support the findings of this study are available on request from the corresponding author. The data are not publicly available due to privacy or ethical restrictions.
